# LncRNA TDRG1-Mediated Overexpression of VEGF Aggravated Retinal Microvascular Endothelial Cell Dysfunction in Diabetic Retinopathy

**DOI:** 10.3389/fphar.2019.01703

**Published:** 2020-01-31

**Authors:** Qiaoyun Gong, Wenpei Dong, Ying Fan, Feng’e Chen, Xiaolan Bian, Xun Xu, Tianwei Qian, Ping Yu

**Affiliations:** ^1^ Department of Ophthalmology, Shanghai General Hospital, National Clinical Research Center for Eye Diseases, Shanghai Key Laboratory of Ocular Fundus Diseases, Shanghai Engineering Center for Visual Science and Photomedicine, Shanghai Engineering Center for Precise Diagnosis and Treatment of Eye Diseases, Shanghai, China; ^2^ Department of General Surgery, Hernia and Abdominal Wall Surgery Center of Shanghai Jiao Tong University, Shanghai Ninth People’s Hospital, Shanghai Jiao Tong University School of Medicine, Shanghai, China; ^3^ Department of Pharmacy, Ruijin Hospital, Shanghai Jiao Tong University School of Medicine, Shanghai, China; ^4^ Department of Ophthalmology, Leiden University Medical Center, Leiden, Netherlands

**Keywords:** LncRNA TDRG1, vascular endothelial growth factor, hyperglycemia, human retinal microvascular endothelial cells, diabetic retinopathy

## Abstract

**Purpose:**

Diabetic retinopathy (DR), a neurovascular disease, is one of the leading causes of blindness in working-age adults. Long noncoding RNAs (lncRNAs) have attracted attention as indicators for DR. This study aimed to characterize the role of lncRNA human testis development–related gene 1 (TDRG1) and its modulation of vascular endothelial growth factor (VEGF) in deteriorating DR.

**Methods:**

Tissue samples were obtained from patients with epiretinal membranes (EMs) or proliferative DR, and human retinal microvascular endothelial cells (HRECs) were cultured with high-glucose medium to mimic DR as the *in vitro* model. The expression of lncRNA TDRG1 and VEGF was determined by immunofluorescence staining, Western blotting, and RT-qPCR. Transfection of small-interfering RNA was conducted to knock down target gene expression. HREC functions were evaluated by cell viability, fluorescein isothiocyanate (FITC)-dextran extravasation, migration, and tube formation assays under different conditions.

**Results:**

LncRNA TDRG1 and VEGF were found to be co-expressed and significantly upregulated in fibrovascular membranes (FVMs) from DR patients compared to those from EM patients. In the *in vitro* model, hyperglycemic treatment markedly increased the expression of lncRNA TDRG1 and VEGF at the mRNA and protein levels, which promoted cell proliferation and migration, enhanced permeability, and disrupted tube formation of HRECs. However, knockdown of lncRNA TDRG1 or VEGF notably decreased the expression of VEGF and reversed the impaired functions of high-glucose-treated HRECs.

**Conclusions:**

LncRNA TDRG1 promoted microvascular cell dysfunction *via* upregulating VEGF in the progression of DR and may serve as a potential therapeutic target in DR treatment.

## Introduction

Diabetic retinopathy (DR) is one of the leading causes of blindness in working-age adults worldwide (Cheung et al., 2010). This type of retinopathy is a severe complication of diabetes mellitus (DM), and its prevalence is increasing dramatically due to the high incidence of DM (Antonetti et al., 2012).In 2010, the prevalence of DM in China was estimated to be 11.6% in the adult population (Xu et al., 2013). Based on a random-effects meta-analysis model in which data on the overall prevalence of DR in China in 2010 were pooled, the pooled prevalence rate of any DR in people with DM was 18.45% (Song et al., 2018). Then, according to a multi-hospital-based DR screening program of patients with diabetes in China from 2014 to 2016, the prevalence of any DR in DM patients was 27.9% (Zhang et al., 2017). The increasing rate of DR in DM patients requires an urgent and effective strategy to target DR as early as possible. Diabetic retinopathy is a chronic microvascular dysfunction that primarily results from long-term detrimental high-glucose exposure (Antonetti et al., 2006). The clinical stages of DR begin with microaneurysm, hemorrhage, cotton wool spot, and lipid exudates, which are non-proliferative characteristics. With the development of poorly controlled DR, proliferative manifestations, including neovascularization, vitreous hemorrhage, fibrovascular membrane (FVM) formation and even tractional retinal detachment, occur (Antonetti et al., 2012; Shin et al., 2014). The pathology of the clinical changes in DR is characterized by retinal microvascular dysfunction that leads to increased vascular permeability, destroyed vascular tubes, and breakdown of the blood-retinal barrier (BRB) (Antonetti et al., 2006). Endothelial cells (ECs) harboring tight junctions are essential for maintaining the inner BRB, and their impairment results in vascular hyperpermeability (Ciulla et al., 2003). Therefore, novel pharmacological therapies targeting biochemical mechanisms that promote EC function may effectively ameliorate DR.

Various vasoactive factors are involved in the microvascular dysfunction and angiogenesis of DR. Notably, vascular endothelial growth factor (VEGF), which mediates the structural and functional changes in the retina and in ECs in diabetic conditions (Kaur et al., 2009; Mima et al., 2012), has attracted considerable attention due to its predominant role in deteriorating the progression of DR. Aberrant expression of VEGF aggravated pathological angiogenesis (Woolard et al., 2004) and then promoted the development of DR (Behl and Kotwani, 2015). Enhanced VEGF induced cellular and retina hyperpermeability, increased cell apoptosis, and promoted cell mobility through multiple signaling mechanisms in DR (Behl and Kotwani, 2015; Gong and Su, 2017). In the past decade, anti-VEGF therapy has been applied to treat DR and other neovascular ocular diseases. However, not all patients are responsive to anti-VEGF therapy, and some show side effects. Thus, other novel therapeutic strategies regulating VEGF are needed for DR treatment.

The role of transcriptional or post-transcriptional regulation is an emerging area of research that is expected to be involved in the pathogenesis of DR. Recent data have revealed that numerous noncoding RNAs (ncRNAs), which have little or no protein-coding potential, are expressed and function in both normal physiology and diseases such as cancers, genetic disorders, and neovascular diseases (Bartel, 2009; Wilusz et al., 2009; Conte et al., 2013). In particular, long ncRNAs (lncRNAs) have been shown to participate in different biological processes, including transcription, post-transcription, translation, epigenetic regulation, splicing, and intracellular/extracellular trafficking (Mercer et al., 2009; Wilusz et al., 2009). LncRNA transcripts are located in the nucleus and cytoplasm, and the transcription of lncRNAs can be modified post-transcriptionally, promoting temporal and cell-type-specific expression (Kapranov et al., 2007; Guttman et al., 2009; van Heesch et al., 2014). LncRNAs may guide transcription factors to, or sequester them from, a specific region of action, or they can interact with various components, thereby suppressing or activating gene expression (Wang and Chang, 2011). Human testis-specific gene testis developmental related gene 1 (TDRG1) is a newly identified lncRNA and encodes a 100 amino acid protein that does not possess any known protein domains (Wang et al., 2016). A previous report showed that lncRNA TDRG1 may enhance the proliferation of bone marrow mesenchymal stem cells *via* fibroblast growth factor 1 (FGF1) (Jiang et al., 2015). Additionally, lncRNA TDRG1 may promote endometrial carcinoma cell proliferation and invasion by positively targeting VEGF-α (Chen et al., 2018). Regarding the potentially important role of lncRNA TDRG1 in angiogenesis, we predicted that it may interact with VEGF and modulate EC functions to affect DR progression. Thus, this study aimed to explore the regulatory effects of lncRNA TDRG1 on VEGF actions in ameliorating DR. LncRNA TDRG1 may be a novel and effective target to inhibit the development of DR.

## Materials and Methods

### Tissue Samples

Tissue samples in this study included the epiretinal membranes (EM) of 12 healthy patients and FVMs of 12 proliferative DR (PDR) patients. All patients were 47–69 years old and received pars plana vitrectomy with membrane peeling. The six membrane specimens surgically obtained from the two groups of patients were fixed in 4% paraformaldehyde, embedded in paraffin and sectioned at 6 μm for H&E staining and immunofluorescence staining. The remaining membranes in each group were extracted and processed with RT-qPCR for RNA detection. All procedures performed in this study involving human participants were in accordance with the 1964 Helsinki Declaration and its later amendments or comparable ethical standards and were approved by the ethics committee of Shanghai General Hospital. Informed consent was obtained from all individual participants included in the study.

### Cell Culture and Treatment

Human retinal microvascular ECs (HRECs) were obtained from ANGIO-PRO TEOMIE (Boston, MA, USA) which were grown on polylysine-coated flask and cultured in EC medium containing 5% fetal bovine serum (FBS), 1% EC growth supplement, and 1% penicillin-streptomycin (ScienCell, Carlsbad, CA, USA) at 37°C in a humidified atmosphere containing 5% CO_2_. HRECs were plated at 1 × 10^4^ cells in 6-well plates (Corning; Acton, MA, USA) and treated with normal glucose (NG; 5.5 mmol/L) as a control or with high glucose (HG; 25 mmol/L) under normoxic conditions for 72 h to mimic the early stage of DR. For maintenance of uniform conditions, the medium was changed daily to eliminate metabolic byproducts and provide the nutrients necessary for the cells, and all of the *in vitro* experiments were carried out using HRECs at passages 3~8.

### Small-Interfering RNA (siRNA) Transfection

Human retinal microvascular endothelial cells in the logarithmic growth phase were seeded in 6‐well plates and exposed to normal or high-glucose medium. After reaching 70%–80% confluence, the cells were transfected with 50 nM siRNA (lncTDRG1 siRNA, VEGF siRNA, or NC siRNA) using Lipofectamine 3000 transfection reagent (Invitrogen, Carlsbad, CA, USA). After transfection for 6 h, fresh high‐glucose medium or normal medium was replaced. The cells were harvested for further mRNA analysis after 48 h, and collected for protein analysis after 72 h. siRNAs were chemically synthesized by GenePharma (Shanghai, China).

### Total RNA Isolation and Quantitative Analysis of mRNAs

Total RNA was extracted from HRECs cultured under different conditions and tissues using a TaKaRa Mini BEST Universal RNA Extraction Kit (TaKaRa Bio, Dalian, China) following the manufacturer’s protocol. The A260/A280 value and concentration were measured by a NanoDrop 2000c Spectrophotometer (Thermo, Waltham, MA, USA). An A260/A280 of approximately 2.0 was generally accepted for further analysis. Then, 500~1,000 ng of total RNA was reverse transcribed into cDNA using a Perfect Real Time RT reagent kit (TaKaRa Bio, Dalian, China) in a 20‐μL reaction volume.

To explore the expression and modulatory mechanism of lncRNA TDRG1 and VEGF in DR, real-time quantitative PCR (RT-qPCR) was performed to measure the levels of lncRNA TDRG1 and VEGF in human membrane samples and HRECs. The qPCR mixture contained 2 μL cDNA, 10 μmol gene‐specific primers (forward and reverse mixed together), and 10 μL of 2 × Fast SYBR Green Master Mix (TaKaRa Bio, Dalian, China). Three replicates for each biological mixture were analyzed on a LightCycler 480 system (Roche Diagnostics). The data were normalized to the expression of the housekeeping gene GAPDH. Primer sequences are listed in **Table 1**. The relative expression levels were calculated using the 2^−ΔΔCt^ method, which was based on the ratio of gene expression between the experimental group and the control group.

**Table 1:** Primer sequences of target genes.

Gene subtype	Oligonucleotide primers (5′–3′)
Human VEGF	F: TTGCTGCTCTACCTCCACCA R: GCTGCGCTGATAGACATCCA
Human lncRNA TDRG1	F: TCTTCCCTGGCTTGGC R: TGGGCTCTTTCGTGGC
GAPDH	F: TGCACCACCAACTGCTTAGC R: GGCATGGACTGTGGTCATGAG

### Western Blotting

To confirm the protein expression and modulatory mechanism of lncRNA TDRG1 on VEGF in DR, total protein was collected from HRECs cultured under hyperglycemic conditions. The cells were lysed for 10 min on ice in radioimmunoprecipitation assay (RIPA) buffer complemented with protease inhibitor phenylmethylsulfonyl fluoride (PMSF, 1 mM, Beyotime, Jiangsu, China) according to the manufacturer’s protocol and then sonicated. The lysates were collected at 12,000 rpm for 10 min at 4°C. Protein concentrations were evaluated using a Bicinchoninic Acid Protein Assay Kit (Thermo, IL, USA). Protein lysates were electrophoresed on 10% SDS polyacrylamide gels, transferred onto polyvinylidene difluoride (PVDF) membranes (Millipore, USA), and then blocked in 5% skim milk for 1 h. Primary antibodies against VEGF (1:1,000, Abcam #ab1316, USA) and GAPDH (1:1,000, CST Signaling #2118, USA) were applied separately at 4°C overnight. Blots were then treated with secondary antibodies (1:5,000; Millipore, USA) for 1 h. Finally, an Enhanced Chemiluminescence (ECL) Plus Kit (Thermo, IL, USA) was applied for visualization. The gray bands were calculated using ImageJ software.

### Immunofluorescence and LncRNA TDRG1 FISH

To investigate the potential roles of lncRNA TDRG1 and VEGF in angiogenesis in the progression of DR, immunofluorescence staining of VEGF and lncRNA TDRG1 was performed using paraffin‐embedded sections or cultured HRECs on poly-lysine‐coated glass coverslips under hyperglycemic conditions. The slides were rinsed, and the cells were fixed with 4% paraformaldehyde, permeabilized with 0.5% Triton X‐100 in PBS for 15 min at room temperature, and blocked (1% bovine serum albumin in PBS) for 1 h at 37°C. Subsequently, the sections were treated with primary rabbit polyclonal anti‐VEGF antibody (1:100, Abcam #ab1316, USA), and RNA fluorescence *in situ* hybridization of lncRNA TDRG1-FISH (1:100, Servicebio, China) was performed at 4°C overnight. Slides were then incubated with Cy3‐conjugated goat anti‐rabbit or goat anti‐mouse IgG DyLight 488‐conjugated secondary antibodies (1:500, CST Signaling, USA) for 1 h at 37°C. Nuclei were stained with DAPI (1:5,000 diluted in PBS; Thermo, IL, USA). The slides were observed under a confocal laser scanning microscope (Leica, Germany). The primer sequence of lncRNA TDRG1-FISH was: 5′-CCTTGCCAGGTAAGTGAAAGTGCGCTCCG-3’. The mean fluorescence intensities were calculated by ImageJ software. Five images for each group in the three independent experiments were evaluated. Specifically, the cell number was included in the calculation process.

### Cell Viability Assessment

Human retinal microvascular endothelial cell viability was assessed by a Cell Counting Kit-8 (CCK-8, Dojindo, Japan) according to the manufacturer’s protocol. HRECs were cultured under hyperglycemic conditions with transfections as described above and then seeded at a density of 2 × 10^3^ cells/well in 96‐well plates. Before detection, 90 μL of fresh medium was replaced, and 10 μL of CCK-8 was added to the cells. After incubation for 1.5 h at 37°C under normal oxygen, the absorbance was measured at 490 nm using a Varioskan Flash system (Thermo, USA). At each time point, the absorbance value was obtained, and then, calculations for each group were performed by comparing the value of each group to that of the responsive negative control (Ctr) group; the value of the control group was set to 1.

### Monolayer Permeability Assay

For evaluating cell permeability, human retinal microvascular endothelial cells treated with different conditions were seeded at 1 × 10^5^ cells/well in the upper chamber (6.5‐mm diameter transwell with 0.4‐μm pore polycarbonate membrane inserts; Corning; USA) and cultured for 48 h to reach confluence. The upper chamber was washed three times with PBS and treated with FITC‐dextran (1 mg/mL; Sigma; USA). The fluorescence intensity, equivalent to the relative amount of FITC‐dextran in the lower chambers of the transwells, was measured over a 3-h incubation at 37°C and determined in triplicate using a Varioskan Flash system (excitation wavelength, 490 nm; emission wavelength, 520 nm; Thermo). The values were collected and calculated *via* comparisons to the responsive ctr group.

### Cell Migration Assay

The migratory ability of HRECs under high glucose was determined using the transwell system. A total of 5 × 10^4^ cells from each group were seeded in the top chambers of 6.5‐mm diameter transwells with 8.0‐μm pore polycarbonate membrane inserts (Corning; USA) and cultured in 100 μL medium with 5% FBS. The bottom chambers were filled with 500 μL medium with 20% FBS. After incubation for 24 h, the cells on the top chamber were removed, and the migrated cells were fixed with 4% paraformaldehyde for 30 min and stained in 0.1% crystal violet solution. Images were captured by microscopy. Cells were counted in five fields for each group and quantification was performed using ImageJ software.

### Tube Formation Assay

The angiogenic formation of HRECs was measured using a tube formation assay. Firstly, 96-well plates were coated with 50 μL of Matrigel Basement Membrane Matrix (BD Biosciences, USA) per well and polymerized for 1 h at 37°C. Then, human retinal microvascular endothelial cells were seeded in the Matrigel-coated plates at 7 × 10^3^ cells per well in 100 μL medium and incubated at 37°C for 6~8 h. The network of tubes was captured by microscopy. Quantification of tube formation was performed using ImageJ software.

### Statistical Analysis

Data are presented as the mean ± SEM from at least three independent experiments. One‐way analysis of variance (one-way ANOVA) was used for multiple comparisons to assess the significant differences between groups. Comparisons between two groups were performed using Student’s *t*-tests (GraphPad Prism 6.0; GraphPad Prism, USA). Two‐sided *p* values < 0.05 were considered statistically significant.

## Results

### Enhanced Coexpression of LncRNA TDRG1 and VEGF in FVMs

**Figure 1:**
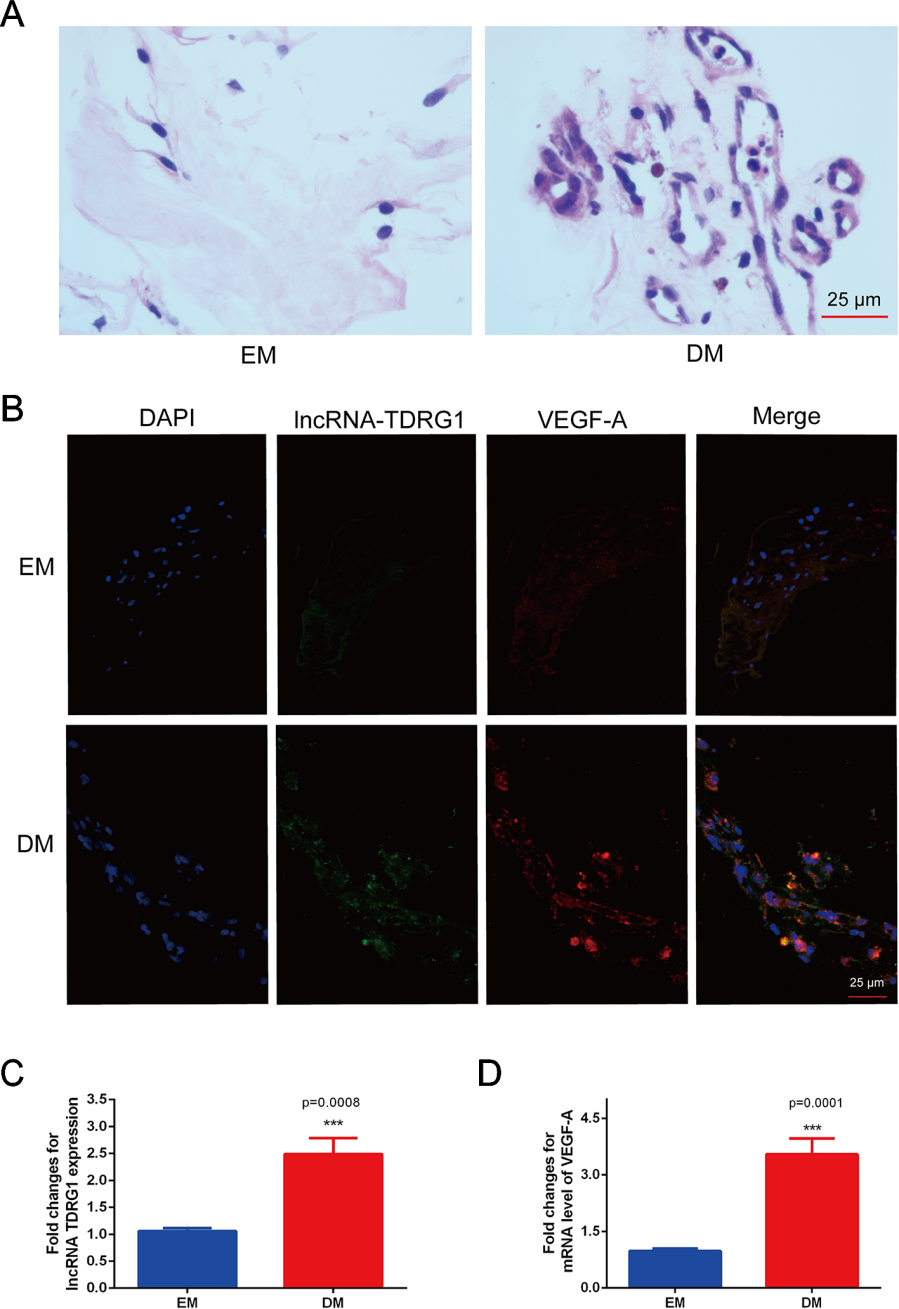
Increased coexpression of lncRNA TDRG1 and VEGF in EMs and FVMs from patients. **(A)** HE staining demonstrated the fibrous connective tissue structure of EMs extracted from patients without DM and FVMs with vascularity from PDR patients with DM. **(B)** Double immunofluorescence staining of lncRNA TDRG1 (green) and VEGF (red) showed that coexpression of lncRNA TDRG1 and VEGF enhanced in the blood vessels of FVMs compared to EMs. **(C, D)** The expression level of lncRNA TDRG1 in FVMs was nearly 3.0-fold higher than that in EMs, while the VEGF mRNA level in FVMs was more than 3.0-fold higher than that in EMs. Bars, mean ± SEM. ***p < 0.001 versus the EM group (n = 6).

The coexpression of lncRNA TDRG1 and VEGF in FVMs from PDR patients and EMs from patients without DM was determined. HE staining showed the pathogenic structure of the EMs and FVMs. EMs had fibrous connective tissue, while FVMs showed both fibers and vascularity (**Figure 1A**). Immunofluorescence staining showed the lncRNA TDRG1 and VEGF were expressed in these two kinds of tissue samples, and high fluorescence was observed in the FVMs, especially in the vessel walls (**Figure 1B**). Moreover, expression of lncRNA TDRG1 and VEGF was increased in FVMs compared to EMs (**Figures 1C, D**), as shown by RT-qPCR and analyzed by Student’s *t*-tests. These results revealed that lncRNA TDRG1 and VEGF were highly coexpressed around the vessels in FVMs at the stage of proliferative DR. Therefore, lncRNA TDRG1 and VEGF might cooperate to deteriorate the progression of DR.

### Upregulated LncRNA TDRG1 and VEGF in HRECs Exposed to High Glucose

**Figure 2:**
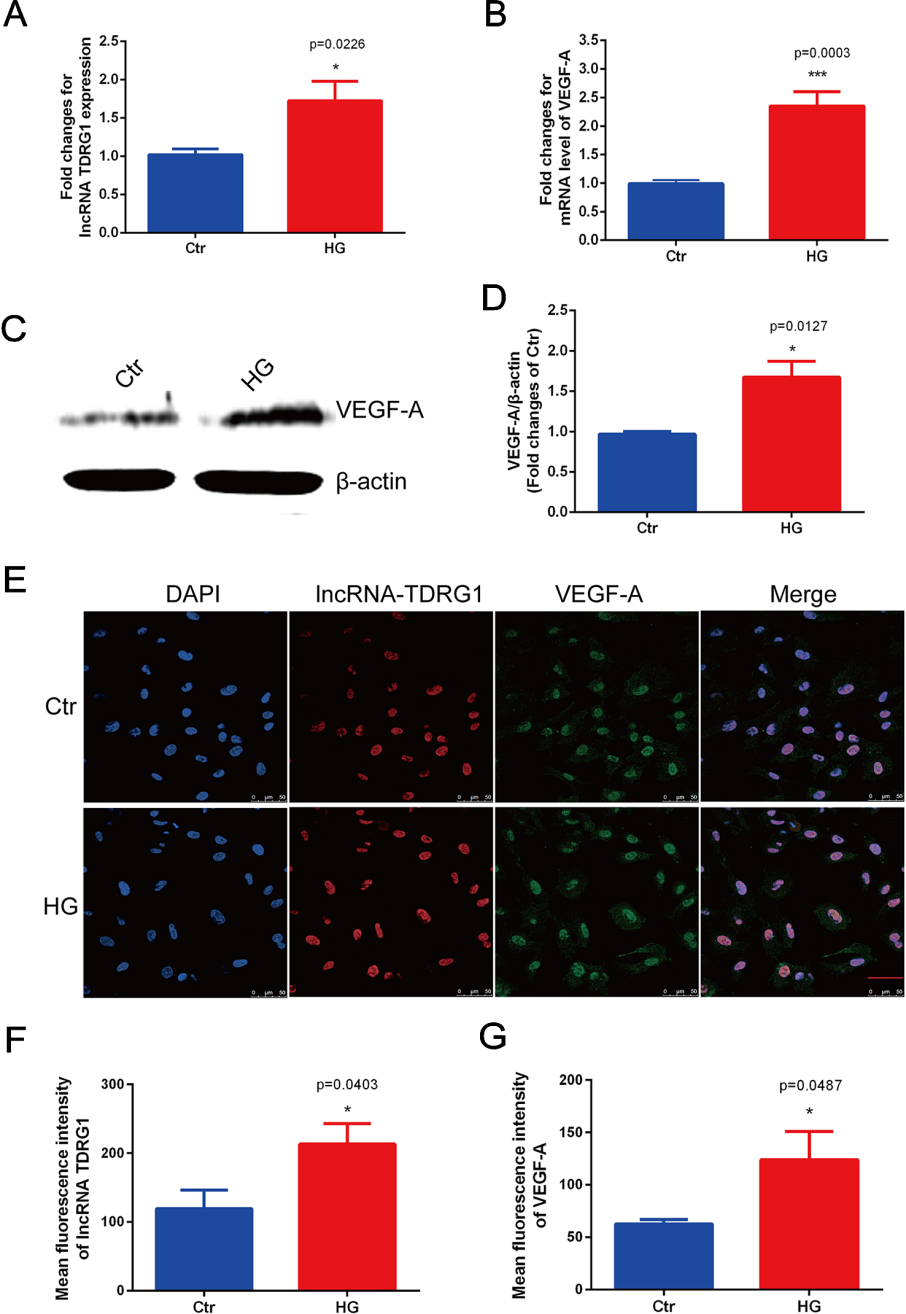
Elevated expression levels of lncRNA TDRG1 and VEGF in HRECs exposed to hyperglycemia. **(A)** RT-qPCR showed that high glucose (HG) induced a 2.0-fold increase in lncRNA TDRG1 in HRECs at the transcriptional level. **(B)** HG significantly upregulated the expression of VEGF at the mRNA level (n = 6). **(C, D)** Western blot analysis demonstrated the protein level of VEGF in HRECs under hyperglycemic conditions. Similar to the transcript variation, VEGF protein was significantly increased after HG treatment (n = 3). **(E)** Dual immunostaining of lncRNA TDRG1 (red) and VEGF (green), merged with DAPI (blue) in HRECs under NG and HG. LncRNA TDRG1 was mainly located in the nucleus, while VEGF was expressed in the nucleus and cytoplasm. The fluorescence was enhanced by hyperglycemia. **(F, G)** Quantitative analysis of fluorescence in lncRNA TDRG1 and VEGF demonstrated a nearly 2.0-fold increase when HRECs were exposed to the HG condition (n = 6). Bars, mean ± SEM. *p < 0.05, ***p < 0.001 versus the negative control group.

Consistent with the *in vivo* results, the mRNA levels of lncRNA TDRG1 and VEGF were enhanced by hyperglycemia in HRECs (**Figures 2A, B**), with observed increases of 2.0-fold in lncRNA TDRG1 and 2.5-fold in VEGF. At the post-transcriptional level, VEGF protein expression was upregulated concomitantly by high glucose (**Figures 2C, D**). Accordingly, lncRNA TDRG1 and VEGF were increased in HRECs in diabetic conditions.

Furthermore, double immunofluorescence staining was performed to determine the orientation and expression of lncRNA TDRG1 and VEGF in HRECs. LncRNA TDRG1 was mainly expressed in the nucleus, while VEGF was detected in the nucleus and cytoplasm in HRECs (**Figure 2E**). The fluorescence of both lncRNA TDRG1 and VEGF under diabetic conditions was notably increased compared to that of the normal group. The quantitative analysis of the fluorescence showed a more than 2.0-fold increase in both lncRNA TDRG1 and VEGF in HRECs under the HG condition (**Figures 2F, G**). These results revealed that the coexpression of lncRNA TDRG1 and VEGF in HRECs could be induced by hyperglycemia. The comparisons between NG and HG were performed using Student’s *t*-tests.

### Positive Regulation of VEGF by LncRNA TDRG1 in Hyperglycemic HRECs

The modulatory relationship between lncRNA TDRG1 and VEGF in DR progression was verified by siRNAs to silence the target genes. The lncTDRG1 siRNA sequences were as follows: sense: 5′-CCUUCCCAGGUCUAGGUUCdTdT-3′; antisense: 5′-GAACCUAGACCUGGGAAGGdTdT-3′. The VEGF siRNA sequences were as follows: sense: 5′-GAAGUUCAUGGAUGUCUAUdTdT-3′; antisense: 5′-AUAGACAUCCAUGAACUUCdTdT-3′.

**Figure 3:**
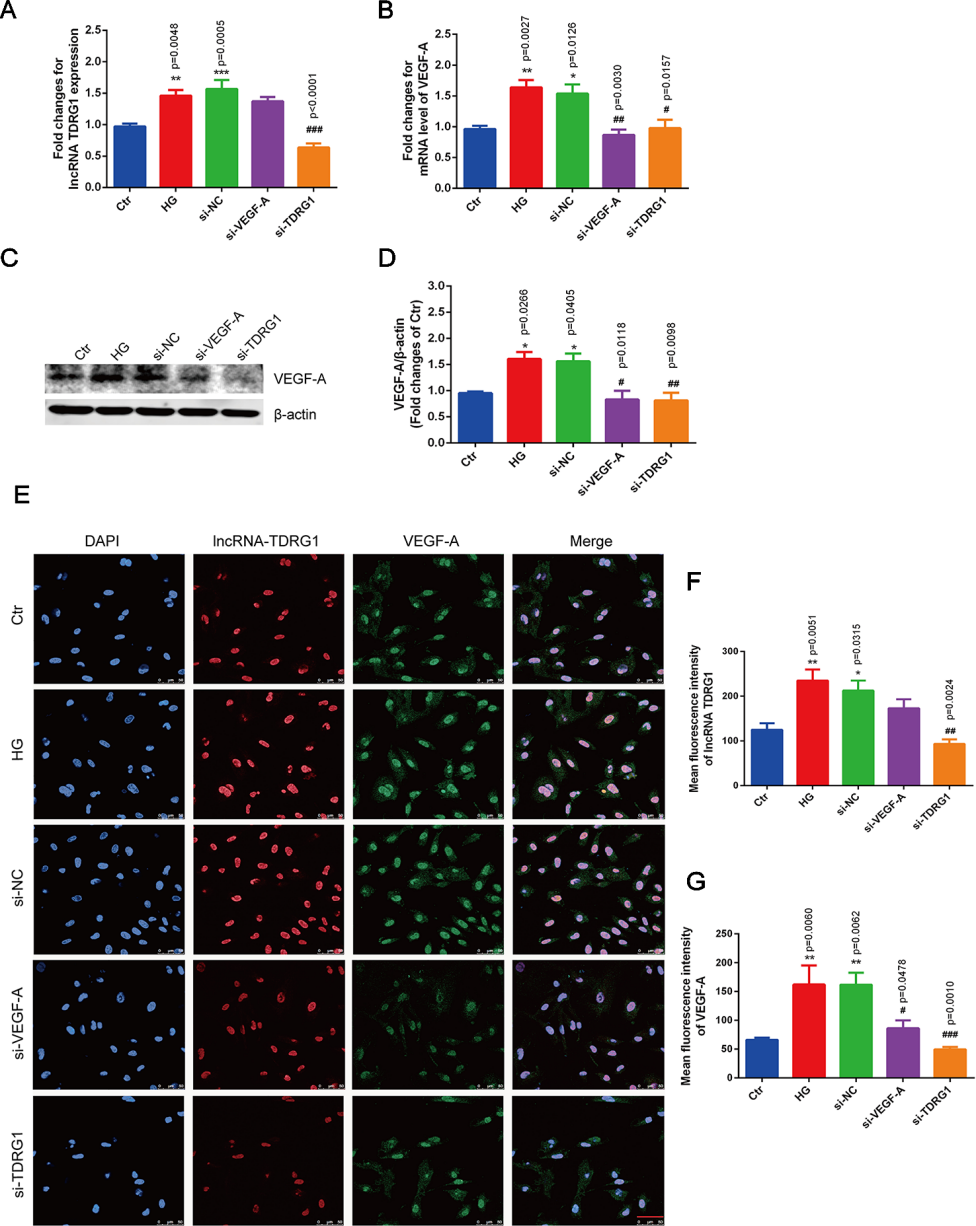
Knockdown of lncRNA TDRG1 reversed the increased expression of VEGF in HRECs under high glucose. **(A)** The mRNA level of VEGF was inhibited by transfection with either VEGF siRNA or lncRNA TDRG1 siRNA. No significant change was observed between the negative transfection and HG groups. **(B)** Expression of lncRNA TDRG1 was suppressed by specific siRNAs in HRECs under HG but was not responsive to VEGF siRNA (n = 6). **(C, D)** The protein level of VEGF was consistent with the mRNA level. β-actin was used as a loading control (n = 4). **(E)** Immunofluorescence and **(F, G)** quantitative analysis revealed that high fluorescence of VEGF in HRECs induced by hyperglycemia could be repressed by knockdown of VEGF and lncRNA TDRG1, while HG-induced fluorescence of lncRNA TDRG1 was suppressed by lncRNA TDRG1 siRNA (n = 5–10). Bars, mean ± SEM. *p < 0.05, **p < 0.01, ***p < 0.001 versus the negative control group; ^#^p < 0.05, ^##^p < 0.01, ^###^p < 0.001 versus the negative control siRNA transfection.

Notably, hyperglycemia upregulated the mRNA levels of lncRNA TDRG1 and VEGF, with an increase of more than 1.5-fold in HRECs, but there was no distinct difference between the high-glucose group and the negative transfection group (**Figures 3A, B**). However, these changes were strongly suppressed by specific siRNAs, and the mRNA levels of lncRNA TDRG1 and VEGF decreased to the level of normal conditions. Moreover, VEGF was repressed following the downregulation of lncRNA TDRG1 in hyperglycemia-induced HRECs. Nevertheless, VEGF knockdown did not affect the expression of lncRNA TDRG1. Correspondingly, the enhanced protein level of VEGF induced by hyperglycemia was inhibited by separate transfection of lncRNA TDRG1 and VEGF siRNAs, while the negative control transfection resulted in no changes (**Figures 3C, D**). Thus, lncRNA TDRG1 positively modulated the expression of VEGF, while VEGF exerted no effects on the lncRNA TDRG1 level.

The changes in the fluorescence intensity of lncRNA TDRG1 and VEGF confirmed the consistent tendency with the mRNA and protein alterations (**Figure 3E**). The fluorescence intensities of lncRNA TDRG1 and VEGF were significantly decreased after transfection of siRNAs targeting lncRNA TDRG1 and VEGF in HRECs under hyperglycemic conditions. The quantitative analysis confirmed that knockdown of lncRNA TDRG1 repressed VEGF expression (**Figures 3F, G**). These data indicated that lncRNA TDRG1 could positively regulate VEGF expression in the progression of DR. One-way ANOVA was used for multiple comparisons to assess the significant differences between groups.

### Effects of LncRNA TDRG1 or VEGF Knockdown on Hyperglycemia-Induced HREC Dysfunction

**Figure 4:**
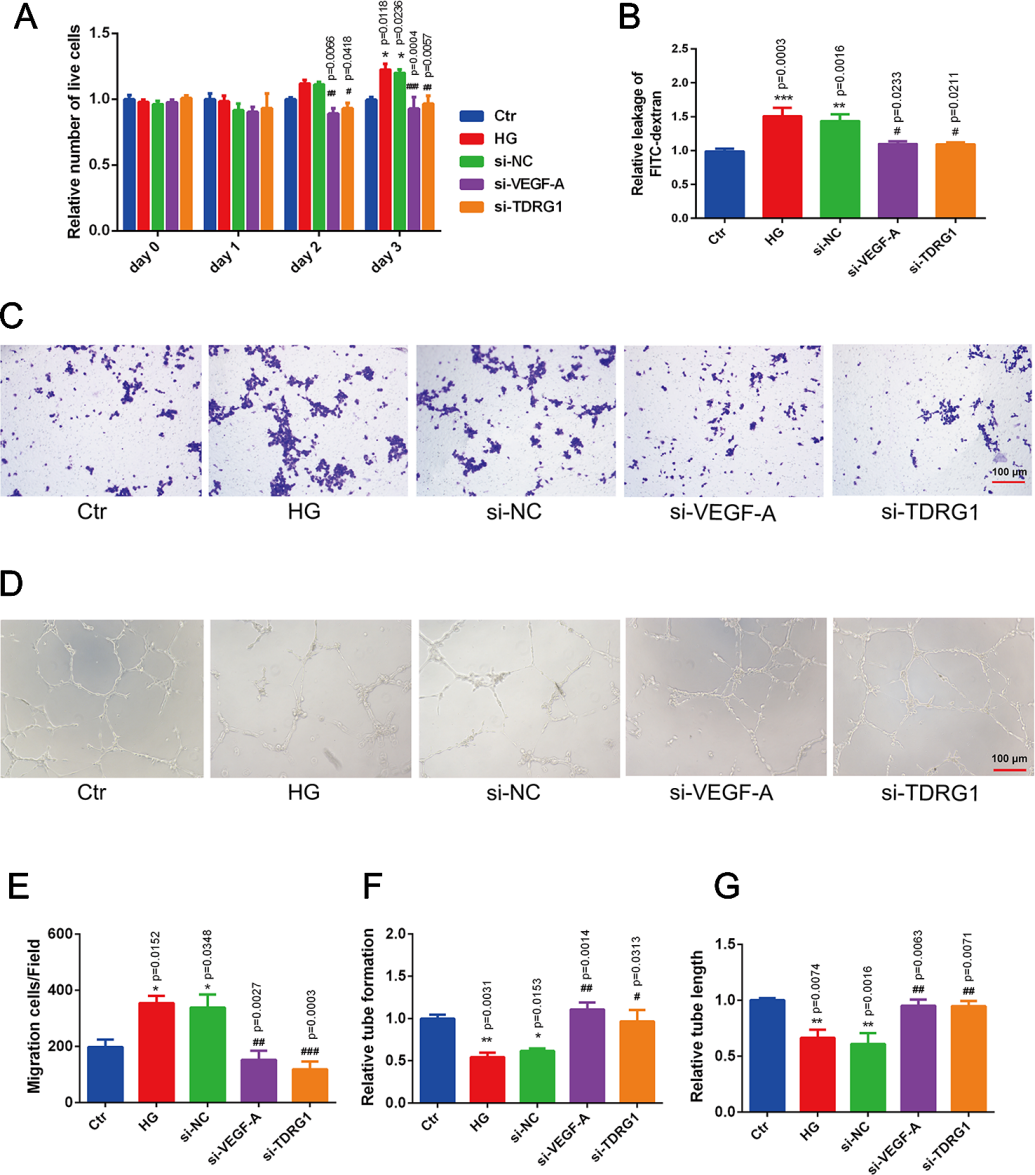
Protective effects of VEGF depletion by lncRNA TDRG1 regulation on HREC dysfunction induced by hyperglycemia. **(A)** Increased proliferation of HRECs caused by HG was reduced by transfection of siRNAs targeting lncRNA TDRG1 and VEGF (n = 5–7). **(B)** Leakage of FITC-dextran demonstrated that hyperglycemia enhanced cell monolayer permeability, whereas this effect was reversed by VEGF or lncRNA TDRG1 knockdown (n = 6). **(C, E)** Increased mitigation of HRECs was observed under DR conditions, and VEGF or lncRNA TDRG1 inhibition exerted the opposite effects (n = 8). **(D, F, G)** Angiogenic ability of HRECs under different conditions. HG destroyed the tube network formation of HRECs compared to the negative control, while VEGF or lncRNA TDRG1 intervention improved the angiogenic ability of HRECs by enhancing tube formation and increasing tubule length. Negative siRNA transfection did not induce any distinct changes in HREC dysfunction. Relative tube formation and tube length were quantified by ImageJ software (n = 6). Bars, mean ± SEM. *p < 0.05, **p < 0.01, ***p < 0.01 versus the negative control group; ^#^p < 0.05, ^##^p < 0.01, ^###^p < 0.001 versus the negative control siRNA transfection.

The pathological changes of diabetic microvascular complications are commonly characterized by abnormally functioning vascular ECs. The effects of lncRNA TDRG1 and VEGF on HRECs in DR conditions were then evaluated. CCK-8 assays showed that knockdown of lncRNA TDRGVEGF could inhibit the accelerated cell proliferation induced by high glucose in ECs compared with that of the negative siRNA-transfected group (**Figure 4A**). A cell permeability assay demonstrated that hyperglycemia enhanced HREC leakage of FITC‐dextran compared with that in the normal group. The monolayer permeability of HRECs was rescued by lncRNA TDRG1 or VEGF downregulation compared with that of negative transfection (**Figure 4B**). The cell migration change under the DR condition was evaluated by transwell assays. The result revealed that diabetic conditions significantly promoted cell migration compared with the control conditions. However, exposure to high glucose, lncRNA TDRG1 intervention or VEGF siRNA could protect against high-glucose‐stimulated HREC migration. The negative control cells under hyperglycemia did not show any significant difference in migration (**Figure 4C**). Both the lncRNA TDRG1 and VEGF interventions resulted in 2.0-fold decreases in migrated cells (**Figure 4E**). Matrigel tube formation was assessed in HRECs cultured under the same conditions as previously described. As demonstrated in **Figure 4D**, diabetic conditions caused a morphological change in HRECs and destroyed the tube network formation compared to the control conditions. In addition, lncRNA TDRG1 intervention distinctly improved the angiogenic ability of HRECs by increasing tube formation and tubule length relative to that of the negative transfection cells in hyperglycemic conditions as well as the effect of VEGF knockdown (**Figures 4F, G**). These results suggested that both lncRNA TDRG1 and VEGF depletion could prevent the breakdown of angiogenesis in HRECs induced by high-glucose conditions. One-way ANOVA was used for multiple comparisons to assess the significant differences between groups.

These findings indicated that modulation of VEGF by lncRNA TDRG1 knockdown had a protective role in HREC viability, cell permeability, motility, and tube integrity under DR conditions. Accordingly, inhibition of VEGF by lncRNA regulation improved HREC morphology in the progression of DR and might be a strategy in clinical therapy.

## Discussion

Diabetic retinopathy is one of the most serious microvascular complications of patients with long-term DM (Cheung et al., 2010; Antonetti et al., 2012), which is pathologically characterized by retinal inflammation, increased permeability, neovascularization, macular edema, and proliferative changes (Bandello et al., 2013). Hyperglycemia is the original and most important factor for diabetes and exerts adverse effects on vascular ECs during the development of microvascular complications (Antonetti et al., 2006). In addition to inflammatory mediators, neural dysfunction, and vascular growth factors, endothelial cells suffer from apoptosis, destroyed tight junctions, increased migration and enhanced permeability, which aggravate the progression of DR (Ciulla et al., 2003; Bandello et al., 2013). Therefore, protecting or reversing EC dysfunction is crucial for ameliorating DR and may be an effective therapeutic strategy.

The pivotal element in the development of DR from the non-proliferative to the proliferative stage is the occurrence and aggravation of neovascularization (NV). Angiogenic and antiangiogenic factors such as VEGF (Behl and Kotwani, 2015), pigment epithelium derived factor (PEDF) (Ibrahim et al., 2015), and angiopoietin (Bento et al., 2010) released by the retina under hyperglycemia act on NV. Most importantly, VEGF is a key stimulator in NV and closely correlates with the development of DR by causing retinal structural and functional dysfunction (Mima et al., 2012; Behl and Kotwani, 2015). Anti-VEGF therapy has been widely used to inhibit NV in retinal diseases that are not limited to DR. Due to the limitations of tolerance, side effects, and undesirable responsiveness of anti-VEGF therapy, development of novel therapeutic targets to modulate VEGF to ameliorate DR and other neovascular retinal diseases is urgently needed.

With advancements in genome-wide analyses of the mammalian transcriptome, the functional significance of lncRNAs has been recognized (Carninci et al., 2005). An increasing number of studies have identified lncRNAs as a new class of modulatory molecules that affect various human diseases by regulating gene expression at the transcriptional, post-transcriptional, or epigenetic level (Wapinski and Chang, 2011; Mercer and Mattick, 2013). Especially in retinal diseases, the change in the abundance of lncRNAs has been assessed and detected by Yan et al. (2014). Altered expression of lncRNAs was identified in the retinas of streptozotocin-induced diabetic mice. Specifically, lncRNA MALAT1 (Liu et al., 2014; Liu et al., 2019), MIAT (Yan et al., 2015), MEG3 (Qiu et al., 2016), and ANRIL (Thomas et al., 2017) were studied *in vivo* in the retinas of diabetic models and *in vitro* in retinal ECs under DR conditions. LncRNA TDRG1 was studied in vascularity in cancers and shown to modulate VEGF-α to aggravate the dysfunction of cancer cells (Wang et al., 2016), which is also a key step in the angiogenesis of DR, indicating the possible involvement of lncRNA TDRG1 in DR. Thus, we paid attention to the important roles of lncRNA TDRG1 in pathological angiogenesis by interacting with VEGF in microvascular diseases, such as DR.

In the present study, we found that lncRNA TDRG1 was strongly expressed in FVMs collected from PDR patients compared to EMs. Simultaneously, VEGF was found to be coexpressed with lncRNA TDRG1 and enhanced in FVMs. Additionally, *in vitro* experiments in hyperglycemic HRECs confirmed the upregulated levels of lncRNA TDRG1 and VEGF. Immunofluorescence showed the coexpression and increase of lncRNA TDRG1 and VEGF in the nucleus of HRECs under DR conditions. Consistent with previous studies (Zhang et al., 2018; Gong et al., 2019), the increased expression of VEGF contributed to the progression of DR. However, to the best of our knowledge, this study shows that high levels of lncRNA TDRG1, which is coexpressed with VEGF, are tightly related to the development of DR. The expression of VEGF was positively regulated by lncRNA TDRG1 in hyperglycemic HRECs. Knockdown of lncRNA TDRG1 significantly suppressed the increased expression of lncRNA TDRG1 and VEGF induced by hyperglycemia, resulting in a level close to the normal level. In a previous study, RIP assays were conducted to confirm that lncRNA TDRG1 binds and targets the VEGF-α protein (Chen et al., 2018). Thus, in DR progression, lncRNA TDRG1 positively activated VEGF. Furthermore, genetic ablation of lncRNA TDRG1 *in vitro* rescued the EC dysfunction resulting from high glucose, including improving cell proliferation, decreasing cell permeability, inhibiting cell migration, and maintaining the integrity of tube formation of HRECs. Therefore, lncRNA TDRG1 could protect EC functions by modulating VEGF and thus ameliorating DR.

However, the limitation of this study is its lack of mechanistic experiments in animal models to confirm the modulatory effects of lncRNA TDRG1 on DR. Because lncRNA TDRG1 is not expressed in mice or rats, an animal model would be established with rabbits. Further *in vivo* studies will be conducted in a diabetic rabbit model, and the modulatory roles of lncRNA TDRG1 in DR will be confirmed in the future.

In conclusion, to the best of our knowledge, we showed that lncRNA TDRG1 and VEGF were coexpressed and enhanced in the FVMs of patients with PDR and hyperglycemic HRECs. Downregulation of lncRNA TDRG1 decreased VEGF expression directly and protected against microvascular cell dysfunction to alleviate the progression of DR. LncRNA TDRG1 may be an effective and novel therapeutic target for the treatment of DR *via* targeting VEGF.

## Data Availability Statement

The datasets used and/or analyzed during the current study are available from the authors on reasonable request.

## Ethics Statement

All procedures performed in studies involving human participants were in accordance with the ethical standards of the Shanghai Jiaotong University and National Research Committee and with the 1964 Helsinki Declaration and its later amendments or comparable ethical standards. Informed consent was obtained from all individual participants included in the study.

## Author Contributions

QG, PY, TQ, XB, and XX conceived and designed the experiments. WD and PY contributed to the acquisition of data. QG and PY analyzed and interpreted the data. YF and FC obtained tissue samples. QG and TQ contributed to drafting the article. All authors have revised the manuscript critically for important intellectual content and approved the final version to be published.

## Funding

This work was supported by the National Science Foundation of China (No. 81800878), the Interdisciplinary Program of Shanghai Jiao Tong University (No. YG2017QN24), the Key Technological Research Projects of Songjiang District (No. 18sjkjgg24), and the Bethune Langmu Ophthalmological Research Fund for Young and Middle-aged People (No. BJ-LM2018002J).

## Conflict of Interest

The authors declare that the research was conducted in the absence of any commercial or financial relationships that could be construed as a potential conflict of interest.

## Abbreviations

DR, diabetic retinopathy; DM, diabetes mellitus; FVM, fibrovascular membrane; BRB, blood-retinal barrier; ECs, endothelial cells; VEGF, vascular endothelial growth factor; lncRNAs, long noncoding RNAs; TDRG1, testis-specific gene testis developmental related gene; HRECs, human retinal microvascular endothelial cells; PDR, proliferative diabetic retinopathy; NG, normal glucose; HG, high glucose; siRNA, small-interfering RNA; EMs, epiretinal membranes; NV, neovascularization.
